# The psychological pathways to maritime English motivation: need satisfaction, frustration, and learning outcomes

**DOI:** 10.3389/fpsyg.2026.1872269

**Published:** 2026-08-24

**Authors:** Xiaoli Hu

**Affiliations:** School of International Education, Jiangsu Maritime Institute, Nanjing, China

**Keywords:** SDT, maritime English, vocational education, basic psychological needs, dual-path model, motivation, PLS-SEM, mixed methods

## Abstract

**Introduction:**

Numerous studies have confirmed the positive effects of Self-Determination Theory (SDT) on educational motivation; however, how its three core psychological needs operate in the high-pressure context of maritime English training remains underexplored. To address this gap, in the present study, we examined the drivers of English-learning motivation among Chinese maritime cadets preparing for Standards of Training, Certification and Watch-keeping for Seafarers (STCW) certification using a non-WEIRD sample.

**Methods:**

A sequential explanatory mixed-methods design was utilized. The quantitative phase tested a dual-path SDT model among 304 cadets, with need satisfaction and frustration as parallel mediators between a need-supportive learning environment and learning outcomes, using Partial Least Squares Structural Equation Modeling (PLS-SEM). The qualitative phase comprised 20 semistructured interviews selected through maximum variation sampling and evaluated using thematic analysis (intercoder reliability: Cohen’s *κ* = 0.84).

**Results:**

Competence satisfaction was the strongest factor for intrinsic motivation (*β* = 0.266, *p* = 0.004), whereas autonomy satisfaction did not have a significant effect (*β* = 0.113, *p* = 0.160). Need frustration was a steady predictor of controlled motivation, with all three frustration types linked to external regulation. The pathway from a supportive environment, through competence satisfaction and identified regulation, to self-perceived learning outcomes was significant (*β* = 0.119, *p* = 0.002). The three qualitative themes—competence seesaw, constrained autonomy, and professional relatedness—showed how certification demands and safety needs contextualize the expression of the three psychological needs in SDT.

**Discussion:**

These findings show that in vocational settings psychological needs are weighed differently. Autonomy support works best as “guided autonomy” within set rules, and relatedness includes connections to the professional community. The dual-path model explains much of the difference between self-reported learning outcomes (R^2^ = 0.744), illustrating that supportive environments help meet needs and lower frustration. This has important implications for maritime English teachers and policymakers who wish to boost motivation in required certification settings.

## 1 Introduction

The international maritime industry is operated by multicultural crews whose safety and operational effectiveness depend on clear, unambiguous communication (Zhang and Cole, 2018; Rom and Khan, 2025). The International Convention on Standards of Training, Certification and Watch-keeping for Seafarers (STCW) requires maritime English as a mandatory requirement for professional certification. Thus, English has become a high-stakes requirement that directly affects cadets’ employability and career advancement. However, in contrast to general education where students can choose their level of engagement, there exists a motivational paradox with maritime vocational education and training (VET) such that while cadets are expected to achieve a prescribed level of skill and knowledge under regulatory pressure, they do not necessarily acquire the internal motivation necessary to actively engage in the learning process (Billett et al., 2020).

This debate extends beyond maritime education. Vocational students have been found to exhibit higher levels of externally regulated motivation and lower levels of intrinsic motivation compared with their academic peers (Zhang et al., 2016; Zhang et al., 2025). Many pursue vocational pathways for pragmatic rather than intrinsic reasons, a distinction that often shapes their motivations and goals and differentiates them from university students (Zhang et al., 2025). Cultural assumptions further complicate this landscape. In academically oriented societies, vocational students frequently encounter negative stereotypes regarding their abilities, which can undermine their academic confidence and persistence (Woronov, 2015; Guo and Wang, 2020). This dynamic is particularly pronounced in China, where unsuccessful performance on the highly competitive Gaokao examination typically results in placement in vocational institutions. Such circumstances can stigmatize students’ educational trajectories before they can demonstrate their capabilities (Zhang and Osman, 2024).

Self-Determination Theory (SDT) is a useful framework to examine these dynamics. SDT states that the quality of motivation depends on whether three basic psychological needs are supported or thwarted: autonomy, competence, and relatedness. Need-supportive environments promote the internalization of extrinsic motivation into more self-determined forms of motivation (Ryan and Deci, 2020). This idea has been robustly supported in many educational settings (Howard et al., 2021), though recent studies in vocational education have demonstrated a more nuanced relationship. For example, Zhang et al. (2025) found that intrinsic and extrinsic goals positively predict well-being among vocational students, challenging the assumption that extrinsic aspirations are inherently detrimental and suggesting a more context-dependent boundary between autonomous and controlled motivation.

The proliferation of generative AI (GenAI) tools in language learning adds a further layer of complexity. For example, Zhang et al. (2026) noted that GenAI-mediated speaking practice improved vocational students’ performance; however, had minimal effect on their intrinsic motivation or self-efficacy. Similarly, Gao et al. (2025) found that vocational students’ writing self-efficacy and critical thinking were unaffected by AI chatbots. These studies demonstrate a pattern where interventions that produce rapid gains in performance often fail to address deeper motivational structures. In a similar vein, Hu (2025) found that interactive feedback mechanisms on social media enhanced adolescents’ perceived competence, autonomy, and relatedness, which in turn fostered intrinsic motivation in English learning—suggesting that when digital tools are designed to support psychological needs, they can positively influence motivation even in informal learning settings. Thus, the mechanisms through which instructional context influences motivational outcomes is likely to be much more context-dependent and much less universal than would be suggested by the broad-brush framework of SDT. Yet none of these studies combined the dual-path SDT model with a mixed-methods design in an STCW-regulated ESP context.

One approach to understanding this dissociation is to examine the mediating role of cognitive demand. Zhang (2024) found that self-efficacy negatively predicted cognitive load among vocational English learners, and that cognitive load distinguished extrinsic from intrinsic motivational pathways. Cheng and Zhang (2026) further examined the serial mediation of self-regulated learning behavior and academic buoyancy in GenAI-assisted Informal Digital Learning of English (IDLE) among low-proficiency vocational students.

Despite these advances, four gaps remain. First, the dual-path SDT model, which considers need satisfaction and frustration as parallel but independent processes, has been validated in general instructional settings (Howard et al., 2017; Olafsen et al., 2021); however, remains untested in high-stakes vocational English. Second, SDT assumes the universal primacy of the three psychological needs (Vansteenkiste et al., 2020). However, the vocational studies reviewed above suggest that context may weight these needs differently—whether competence assumes primacy over autonomy in mandatory, safety-critical training remains an empirical question. Third, the motivational mechanisms linking need-supportive environments to learning outcomes in maritime VET have not yet been examined using a mixed-method design that entails statistical pathways and cadets’ lived experiences. Fourth, and most critically, no study has explicitly examined the dual-path SDT model in maritime vocational English—a context characterized by international safety regulations, multicultural crew communication expectations, and externally imposed certification that differs substantially from other vocational domains such as tourism or business.

To address these research gaps, we employed a sequential explanatory mixed-methods design to investigate English-learning motivation among Chinese maritime cadets. We used a quantitative component, consisting of in-depth interviews that provide contextual insights into cadets’ experiences, to test the dual-path SDT model, in which need satisfaction and frustration function as parallel mediators between a need-supportive environment and learning outcomes. This study was guided by three research questions: (1) How do need satisfaction and frustration operate as parallel mediators between a need-supportive environment and learning outcomes? (2) Which of the three psychological needs is the strongest predictor of self-determined motivation under the pressures of vocational training? (3) What do cadets’ lived experiences reveal about the observed quantitative patterns, particularly regarding the differential weighting of psychological needs in the context of certification requirements? Overall, this study aimed to address gaps among STCW-bound cadets by capturing statistical pathways and shared experiences and by extending prior GenAI work into a new certification-bound context using a mixed-methods study design.

## 2 Literature review and hypothesis development

### 2.1 Self-determination theory: an overview

SDT is a macro-theory of human motivation that distinguishes between the quality and quantity of motivation (Ryan and Deci, 2017). Unlike approaches that treat motivation as a unitary construct varying primarily in intensity, SDT proposes that motivation varies along a continuum of autonomy, the degree to which behavior is self-endorsed rather than externally controlled. At one end lies motivation, a state of non-intention or lack of engagement. At the other end lies intrinsic motivation, characterized by engagement driven by inherent interest and enjoyment. Between these extremes lie four forms of extrinsic motivation—external, introjected, identified, and integrated regulation—which differ in the extent to which the individual has internalized the value of the activity (Ryan and Deci, 2020).

The SDT is based on the premise that every person has three basic psychological needs. Individuals need to feel as if they act freely and with a sense of choice (autonomy). They also need to feel effective and efficacious in what they do (competence). Finally, they need to feel important to others, and that others are important to them (relatedness). When these needs are met, motivation flourishes, and well-being follows; when they are thwarted, motivation suffers, and negative outcomes emerge (Ryan and Deci, 2017; Vansteenkiste et al., 2020). This specific pattern has been observed across a broad range of educational settings in a large body of meta-analytic research (Howard et al., 2021).

However, this universalist picture has been complicated by recent studies in vocational education contexts. Zhang et al. (2025) similarly observed that vocational students’ well-being was positively associated with both internal and external goals, challenging the assumption that extrinsic aspirations are inherently detrimental—a pattern consistent with a more context-dependent reading of SDT. These findings suggest that in vocational settings, where passing certification exams and finding employment are critical, the relationship between autonomous and controlled motivation may be less definite than the universal SDT model allows. Zhang (2024) further demonstrated a bifurcation in vocational learning contexts: cognitive load fully mediated the self-efficacy to extrinsic motivation pathway, but not the intrinsic motivation pathway.

Recent studies in technology-enhanced vocational learning have demonstrated a consistent dissociation between performance gains and motivational change. Zhang et al. (2026) found that GenAI-mediated speaking practice improved performance but not intrinsic motivation, suggesting that motivational mechanisms operate independently of skill gains—directly informing our expectation that competence satisfaction, rather than performance, drives autonomous motivation. Similarly, Gao et al. (2025) found that AI chatbot-integrated writing tasks yielded performance gains without corresponding improvements in self-efficacy. Together, these findings support the view that vocational settings actively shape the relationships SDT presumes.

Cheng and Zhang (2026) found that GenAI-IDLE activities positively predicted autonomous learning behavior and academic buoyancy, substantially reducing L2 demotivation through a chain-mediated mechanism. This logic—need-supportive activities operating through serial behavioral and psychological pathways—parallels our hypothesized serial mediation chain from need satisfaction through identified regulation to learning outcomes.

The theoretical framework for our investigation was based on SDT, specifically the dual-path model, which proposes that need satisfaction and need frustration are parallel but independent processes (Howard et al., 2017; Olafsen et al., 2021). This model supposes that both processes are not merely at the opposite ends of a single continuum but instead act as independent dimensions that coexist, each with distinct drivers and consequences (Chen et al., 2015).

### 2.2 Need-supportive learning environment

A need-supportive learning environment is one in which instructional settings are purposefully designed to satisfy students’ basic psychological needs of autonomy, competence, and relatedness (Ryan and Deci, 2020). Practically, this means that teachers offer meaningful choices and respecting students’ perspectives, provide challenges that are neither too difficult nor too easy, deliver positive feedback, and exhibit genuine warmth and care (Reeve and Cheon, 2021). Together, these practices meet the basic psychological needs: of supporting autonomy, building competence, and nurturing relatedness.

The methods used to measure the need-supportive learning environment construct have varied considerably, depending on the context and goals of the study. The best-known method is the Learning Climate Questionnaire (LCQ; Ryan and Deci, 2020), which measures the extent to which students perceive their teachers as supporting their autonomy. Chiu (2021) adapted this instrument for online settings and demonstrated that it remains a strong predictor of student engagement even when learning is conducted outside the physical classroom. Regarding second language acquisition literature, need-supportive environments tend to foster autonomous motivation and actual language performance (Noels et al., 2019; Alamer, 2021). In vocational education, Zhang et al. (2025) provide a particularly instructive finding—need support was related to well-being, but only indirectly, through its shaping of motivational regulation. This implies that the quality of the learning environment affects students’ feelings and performance, even in the presence of external incentives, such as certification and job prospects.

In high-stakes vocational programs such as maritime English training, the nature of need-supportive environments is relatively different from that found in general education. Traditional academic teachers frequently have considerable freedom in shaping their courses, whereas maritime education teachers are constrained by a tight framework of STCW-prescribed content and certification requirements. This external constraint produces an internal tension: how can instructors address students’ psychological needs when the syllabus itself is mostly non-negotiable? One solution has been to provide options regarding how students learn by employing different teaching methods, and by modifying the sequence and pace of the curriculum. When educators are successful, they satisfy students’ basic needs; in so doing, they also promote autonomous motivation even when external limits are imposed.

### 2.3 Basic psychological need satisfaction and frustration

SDT distinguishes between need satisfaction (the experience of having one’s needs fulfilled) and need frustration (the experience of having one’s needs actively thwarted; Chen et al., 2015). These are empirically separable dimensions rather than opposite poles of a single continuum. Need satisfaction promotes autonomous motivation, well-being, and positive learning outcomes, whereas need frustration predicts controlled motivation, ill-being, and maladaptive learning behaviors (Vansteenkiste et al., 2020).

The Basic Psychological Need Satisfaction and Frustration Scale (BPNSFS), validated by Chen et al. (2015) across four cultures, comprises six subscales measuring satisfaction and frustration with autonomy, competence, and relatedness. The BPNSFS has proved to be a versatile instrument across several studies and has recently been incorporated into vocational education research. For example, Gao et al. (2025) applied the BPNSFS to study AI chatbot-integrated writing tasks among vocational students and found that need satisfaction was a more consistent predictor of positive learning outcomes than the absence of frustration.

The dual-path model has been previously validated in employment and instructional settings (Howard et al., 2017; Olafsen et al., 2021), showing that need satisfaction and frustration have distinct predictive pathways. However, its application to high-stakes vocational English contexts remains limited. In situations where external certification mandates are non-negotiable, the relative importance of different needs—and whether satisfaction and frustration operate through the same mechanisms—remains an empirical question. Cheng and Zhang (2026) found that among low-proficiency vocational students, the protective effects of need-supportive IDLE activities operated using sequential pathways via autonomous learning behavior and academic buoyancy, suggesting that multiple mechanisms mediate the effects of need support on motivational outcomes.

### 2.4 Motivational regulations: autonomous and controlled motivation

According to SDT, the types of external motivation are differentiated by the degree of internalization. External regulation involves behavior driven by external contingencies such as rewards or punishments. SDT distinguishes between three types of extrinsic motivation along a continuum of increasing autonomy. Introjected regulation is located at the controlled end of the continuum and is driven by internal pressures such as guilt, shame, or the quest for contingent self-esteem. Further along the continuum is identified regulation, in which behavior is guided by values and goals personally endorsed by the individual. Integrated regulation is at the most autonomous end of the spectrum, where activities originating outside the individual are made fully congruent with the individual’s larger sense of self (Ryan and Deci, 2020). In the present study, we focus on intrinsic motivation and identified regulation as the two key indicators of autonomous motivation, following common practice in SDT research where integrated regulation is often not separately assessed due to its close empirical overlap with identified regulation in educational contexts (Vansteenkiste et al., 2020; Howard et al., 2021).

Given their conceptual proximity, these regulation types are often separated into two higher-level composites for analytical purposes. Autonomous motivation includes intrinsic motivation, integrated regulation, and identified regulation, while controlled motivation includes introjected regulation and external regulation. This dichotomous classification preserves the important theoretical distinction between self-endorsed and externally pressured motivations and provides analytical parsimony (Howard et al., 2021). The two-factor structure has demonstrated good generalizability across different educational settings and has recently been found to apply to vocational education (Zhang et al., 2025).

The autonomous-controlled distinction is not merely a theoretical nicety, but one that matters for actual student performance and persistence. This is supported by the results of meta-analyses, which show that autonomous motivation generally benefits achievement, engagement, and persistence, whereas controlled motivation tends to yield small or even negative effects (Howard et al., 2021). Interestingly, this relationship is more complex in vocational settings. In a study by Zhang (2024) in vocational English learners, cognitive load fully moderated the relationship between self-efficacy and extrinsic motivation, but not the intrinsic motivation link. This disparity shows that separate psychological mechanisms underpin the two motivational types. Zhang et al. (2025) found that among Chinese vocational students, autonomous and controlled motivation were both positively associated with well-being (with autonomous motivation being more important), and motivation served as a mediator and a moderator between goals and well-being. Altogether, these findings suggest that controlled motivation may not be uniformly detrimental in high-stakes vocational settings where certification is non-negotiable, such as the maritime English STCW certification, particularly when students perceive value in achieving their professional goals.

### 2.5 English learning outcomes

The learning outcomes in this study were operationalized as self-reported perceived improvement and engagement in maritime English learning. An 8-item scale was used to measure learning outcomes. Self-reported measures were particularly appropriate for the current investigation as they capture cadets’ perceived communicative confidence and classroom engagement, dimensions that are critical to sustained motivation in vocational contexts but often overlooked by proficiency exams (Gan et al., 2023). This outcome construct was used as the outcome variable in the model, theorized to be predicted by autonomous motivation and not influenced by controlled motivation.

Vocational English learning results include not just language proficiency but career communication skills, confidence in using English professionally, and active engagement in professional identity-building activities (Rom and Khan, 2025; Guo et al., 2026) as well. Measuring vocational English learning requires assessing more than maritime cadet test scores and pass rates; one must also determine the student’s calmness and ability to communicate in multilingual crews during stressful operations. This larger view of outcomes, which emphasizes professional identity, aligns with vocational education experts’ conception of meaningful learning (Billett et al., 2020).

The dissociation between performance gains and motivational change, consistently observed across studies (Zhang, 2024; Gao et al., 2025; Zhang et al., 2026), underscores the need for outcome measures that capture objective performance and psychological engagement.

### 2.6 Hypothesis development

#### 2.6.1 Pathway 1: need satisfaction into autonomous motivation

SDT assumes that need-supportive environments promote the satisfaction of the three basic psychological needs (Ryan and Deci, 2020). This relationship has been confirmed across educational contexts, including vocational education (Chen et al., 2015; Howard et al., 2021). Need satisfaction, in turn, is theorized to foster autonomous motivation, including intrinsic motivation and identified regulation. In second language acquisition contexts, need satisfaction has been shown to predict stronger autonomous motivation (Noels et al., 2019; Alamer, 2021). Specifically in vocational contexts, Cheng and Zhang (2026) found that need-supportive GenAI-IDLE activities positively predicted autonomous learning behavior among low-proficiency vocational students.

Thus, the following hypotheses are proposed:

*H*1a–*H*1c: A need–supportive English learning environment positively predicts maritime cadets’ perceived satisfaction of autonomy, competence, and relatedness.

*H*2a–*H*2c: Perceived autonomy satisfaction, competence satisfaction, and relatedness satisfaction are positively associated with autonomous motivation (intrinsic motivation and identified regulation).

*H*3: Perceived competence, autonomy, and relatedness satisfaction mediate the relationship between a need-supportive environment and intrinsic motivation.

*H*4: Intrinsic motivation positively predicts self-reported English learning outcomes.

However, the relationship between a need-supportive environment and identified regulation may not be straightforward in mandatory vocational contexts, where identified regulation is already high. As Zhang et al. (2025) found, vocational students may have already internalized the value of their learning due to pragmatic career considerations, yielding less space for additional need support to further increase identified regulation. The qualitative findings of Cheng and Zhang (2026) similarly suggest that students with low English proficiency may develop academic buoyancy and autonomous learning behavior with support; however, the ceiling effect in identified regulation may attenuate the direct effects of need support on identified regulation. Nevertheless, based on SDT’s theoretical estimates:

*H*5: A need-supportive environment positively predicts identified regulation.

*H*6: The relationship between a need-supportive environment and English learning outcomes is mediated sequentially by need satisfaction (competence, relatedness, and autonomy) and identified regulation.

#### 2.6.2 Pathway 2: need frustration into controlled motivation

SDT implies that need-supportive environments reduce need frustration, and that need frustration promotes controlled forms of motivation (external and introjected regulation) rather than autonomous forms (Ryan and Deci, 2017; Vansteenkiste et al., 2020). In an educational context, need frustration has been shown to predict external and introjected regulation, which in turn predict negative or null learning outcomes (Howard et al., 2021). Support for this prediction comes from vocational education research suggesting that controlled motivation may not be uniformly detrimental when external goals are salient (Zhang et al., 2025; Gao et al., 2025).

Thus, the following hypotheses are proposed:

*H*7a–*H*7c: A need-supportive English learning environment negatively predicts maritime cadets’ perceived frustration of autonomy, competence, and relatedness.

*H*8a–*H*8c: Perceived autonomy frustration, competence frustration, and relatedness frustration are positively associated with controlled motivation (external regulation and introjected regulation).

*H*9a: External regulation has no statistically significant positive effect on English learning outcomes.

*H*9b: Introjected regulation negatively predicts English learning outcomes.

**Figure 1:**
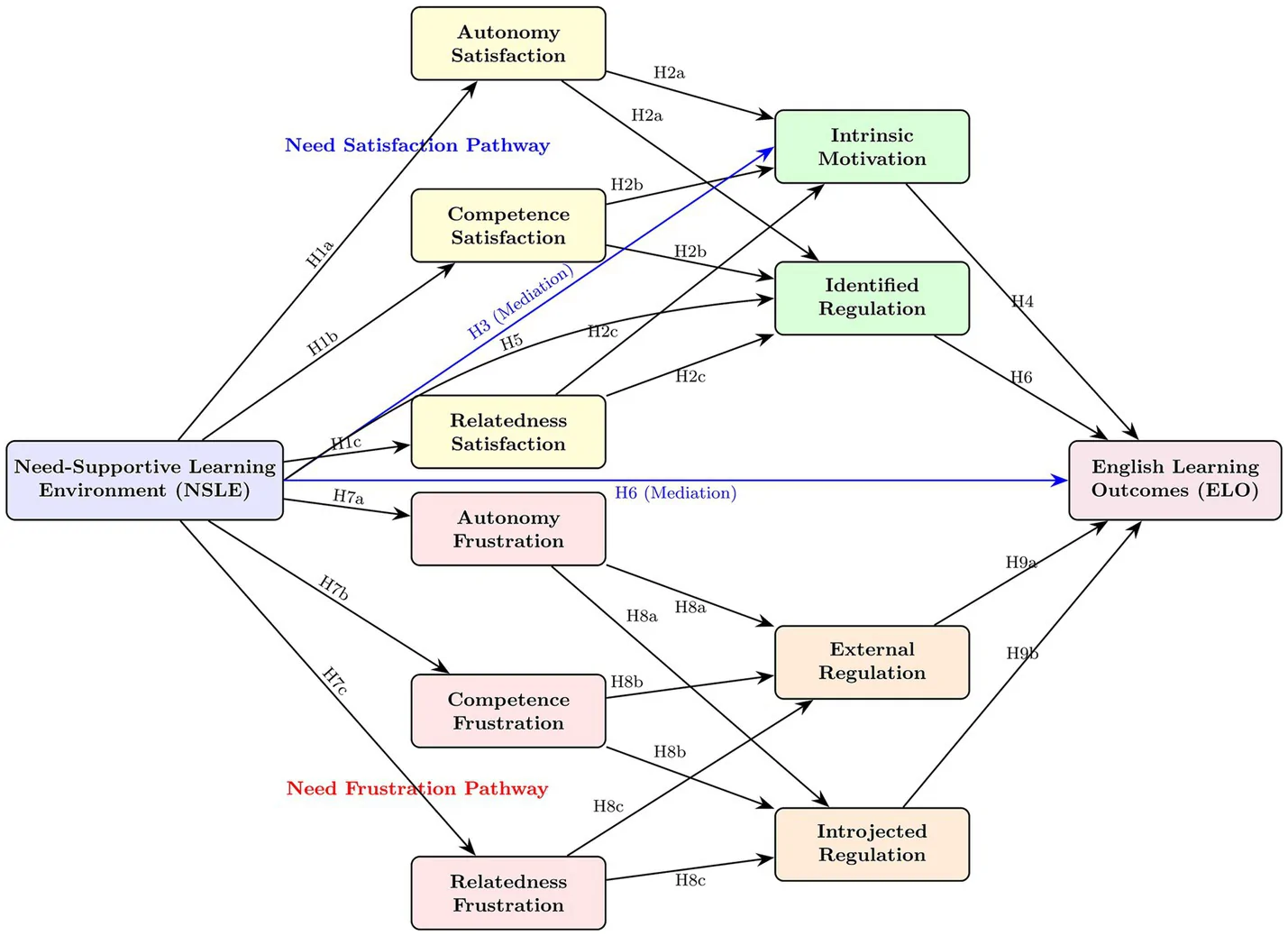
Proposed research model.

The hypothesized relationships are visually summarized in Figure 1.

## 3 Methods

### 3.1 Research design

This study adopted an explanatory sequential mixed-methods design (Robinson, 2007; Creswell and Plano Clark, 2018), in which the primary quantitative phase was followed by a complementary qualitative phase designed to elucidate and explain the initial quantitative findings. This design is particularly appropriate when seeking to use qualitative data to interpret and deepen understanding of statistical patterns, particularly when unexpected or contextually specific results emerge (Tashakkori and Teddlie, 2010).

In the quantitative phase, we surveyed a diverse group of maritime cadets to test our proposed research model. Following survey, we conducted in-depth interviews with a carefully selected subset of respondents. Our second step was to capture the cadets’ English learning experiences beyond the questionnaire.

### 3.2 Participants and sampling

#### 3.2.1 Quantitative phase

The study was conducted at a maritime vocational college in Eastern China. To ensure that our quantitative sample was representative of the diversity of the student population, we used a stratified random sampling procedure, with proportional representation across majors and year levels (Hair et al., 2019). A total of 472 surveys were distributed. We applied our data-cleaning criteria (see Section 3.5.1) and retained 304 valid responses for the final analysis. This gave us a valid response rate of 64.4%, which we consider adequate for the structural equation modeling that followed (Table 1).

**Table 1:** Demographic details of the participants.

Demographic variable	Category	Frequency (*N* = 304)	Percentage (%)
Gender	Male	230	75.7
	Female	74	24.3
Year of study	Year 1	129	42.4
	Year 2	124	40.8
	Year 3	51	16.8
Major	Navigation	87	28.6
	Marine Engineering	111	36.5
	Marine Electro-Technology	106	34.9

Participants ranged in age from 18 to 34 years, with a mean age of 21.4 years (standard deviation [SD] = 2.8 years). The sample was predominantly male (*n* = 230, 75.7%), consistent with the typical gender distribution of maritime programs in China, and students had participated in 1, 2, or 3 years of study (42.4% first-year students, 40.8% second-year students, and 16.8% third-year students). The sample includes cadets from major programs, including Navigation (28.6%), Marine Engineering (36.5%), and marine electro-technology (34.9%).

#### 3.2.2 Qualitative phase

In the qualitative phase, we selected 20 cadets from the survey respondents using a maximum variation sampling strategy (Patton, 2015). Participants were selected based on their composite scores of Intrinsic Motivation (IM) and Identified Regulation (IR), the two main motivational constructs in our quantitative model.

To capture the full range of motivational profiles, all respondents were ranked based on their IM + IR composite scores (mean = 8.17, SD = 1.19), and individuals were recruited from three strata: upper 25% (score ≥ 8.96, *n* = 7), lower 25% (score ≤ 7.38, *n* = 7), and middle 50% (scores between 7.39 and 8.95, *n* = 6).

This stratification meant that our qualitative sample was representative of the motivational continuum seen in the larger survey. Additionally, we balanced the sample across year of study (Year 1: *n* = 7; Year 2: *n* = 7; Year 3: *n* = 6) and program (Navigation: *n* = 6; Marine Engineering: *n* = 7; marine electro-technology: *n* = 7) to capture diverse educational experiences. All 20 selected cadets agreed to participate and signed written informed consent.

### 3.3 Instruments

#### 3.3.1 Quantitative instruments

For the quantitative strand, we used Wenjuanxing (WJX), a Chinese online survey platform that automatically records response completion time and IP address. Questions were separated into two broad sections: one covering demographic and background information, and the other comprising the adapted scales for our core variables. All items used a 5-point Likert scale—from 1 (Strongly Disagree) to 5 (Strongly Agree)—a format our participants were previously familiar with. To adapt the scales to a Chinese-specific context, we followed a translation and back-translation procedure (Brislin, 1970). We then trialed the preliminary version to 30 cadets, verifying that the wording was clear and that the scales produced consistent responses before they were provided to the full cohort.

Students’ perceptions of autonomy support were measured using a 9-item scale drawn from the LCQ (Ryan and Deci, 2020; Chiu, 2021). This scale records the level of freedom students experience in their English classes (e.g., “My English teacher provides me with choices and options”). We also included items that capture whether teachers acknowledge students’ viewpoints, offer meaningful justifications for the tasks they require students to perform, or encourage learners to take initiative for their own learning.

*Basic Psychological Need Satisfaction and Frustration*. To capture how cadets experience the support and frustration of their psychological needs, we adapted the 12-item BPNSFS used by Chen et al. (2015) for the maritime English classroom. The scale maps six dimensions—three representing need satisfaction (autonomy, competence, and relatedness) and three representing need frustration on the same fronts—with two items assigned to each. Satisfaction-related statements were phrased as such: “I feel free to express my ideas in English class” (autonomy), “I feel confident that I can do well in English learning” (competence), and “I feel close and connected with other students in my English class” (relatedness). Parallel frustration items were similarly worded with opposing statements.

Chen et al. (2015) originally validated this version of the 12-item BPNSFS alongside the full 24-item scale and demonstrated adequate psychometric properties across four cultural contexts. Subsequent independent validation studies, including recent work by Heissel et al. (2023), have further supported its six-factor structure and reliability. We adopted the abbreviated version to strike a balance between measurement breadth and participant burden; the full 24-item scale would have considerably extended the overall questionnaire, which already included multiple instruments. Reliability and validity statistics for all scales in our sample are reported in Table 2.

**Table 2:** Reliability and validity statistics.

Construct	Cronbach’s α	ρa	ρc	AVE
NSLE	0.969	0.969	0.970	0.804
Autonomy Satisfaction	0.895	0.896	0.896	0.905
Autonomy Frustration	0.965	0.965	0.965	0.966
Competence Satisfaction	0.898	0.899	0.899	0.908
Competence Frustration	0.947	0.948	0.948	0.950
Relatedness Satisfaction	0.946	0.946	0.946	0.949
Relatedness Frustration	0.970	0.973	0.973	0.971
External Regulation	0.724	0.727	0.727	0.784
Introjected Regulation	0.844	0.931	0.931	0.759
Identified Regulation	0.928	0.929	0.929	0.823
Intrinsic Motivation	0.970	0.970	0.970	0.917
English Learning Outcomes	0.967	0.967	0.967	0.812

Following Chen et al. (2015), we treated need satisfaction and need frustration as separate, though related, dimensions. In utilizing a partial least squares structural equation model (PLS-SEM), the three satisfaction subscales serve as reflective indicators of a higher-order need satisfaction construct, and the three frustration subscales similarly form need frustration. This specification allowed us to examine both pathways simultaneously while preserving their conceptual distinction.

*Motivation Types*. To assess students’ motivational regulations, we used the Academic Self-Regulation Questionnaire (SRQ-A; Ryan and Connell, 1989), which we adapted to English learning. The scale captures the full range of motivational quality, from being forced by others to being fully self-motivated. External regulation (“I study English because I have to pass the mandatory maritime English exams”) and introjected regulation (“I study English because I would feel ashamed if I failed”) are considered controlled motivation. Further on the continuum toward autonomy are identified regulation (“Learning English is important to me for my future career as a seafarer”) and intrinsic motivation (“I learn English because I enjoy it”), which reflect self-endorsed forms of engagement (Ryan and Connell, 1989; Tian et al., 2025).

*English Learning Outcomes (ELO)*. Drawing from previous research in second language acquisition (SLA; e.g., Gan et al., 2023), we created an 8-item self-report scale to measure students’ perceived learning progress and engagement. The items cover different parts of the English learning experience, including vocabulary, listening, speaking, confidence in using maritime English, and class engagement. The purpose of this instrument was to gage cadets’ subjective perceptions of their learning outcomes. The full questionnaire is provided in Appendix A.

#### 3.3.2 Qualitative instrument

To complement the quantitative results, we conducted semistructured interviews of a subset of cadets, designed to explore their subjective learning experiences in depth. The interview protocol was iteratively developed, based on insights from the quantitative findings and the core tenets of SDT, to ensure that the questions would explore participants’ perceptions of their learning environment, their motivational orientations, their experiences of psychological need satisfaction and frustration, and the influence of certification requirements on their English learning. The full protocol (10 open-ended questions) is available in Appendix B.

The following representative questions illustrate the nature of the inquiry:

“Think about a time in your English class when you felt particularly capable and successful. What was happening? What did the teacher or your classmates do?”

“How much influence do you feel you have over what you learn in English class (e.g., topics) and how you learn it? Can you give examples of choices you have or wish you had?”

“You mentioned your future career. Can you describe how learning English feels connected to becoming a seafarer? Does this connection make learning feel more meaningful or more like pressure?”

Interviews ranged from approximately 30 to 45 min in length. All sessions were audio-recorded with participants’ consent and transcribed verbatim. All interviews were conducted in Chinese so that participants could speak freely and clearly, and transcripts were translated into English for analysis and reporting.

### 3.4 Procedure

Ethical approval was obtained from the institutional review board prior to data collection. All procedures adhered to the ethical guidelines outlined in the Declaration of Helsinki. Informed consent was obtained from all participants, who were informed of their right to withdraw from the study at any time without consequence. Survey responses were collected confidentially. For those who consented to follow-up interviews, contact email addresses were collected separately from the main survey data, and participants were assured that their responses and identities would remain confidential.

**Quantitative data collection**. For the survey, an invitation with a link to the anonymous online questionnaire was distributed through class advisors. Data collection lasted for 2 weeks. Participants were assured that their responses would be used only for research purposes and would remain confidential.

**Qualitative data collection**. Following preliminary analysis of the quantitative data, potential interview participants were contacted via email using the sampling strategy described in Section 3.2.2. Interviews were conducted in a private campus office or via a secure video-conferencing platform.

### 3.5 Data analysis

#### 3.5.1 Data cleaning and preliminary analysis

Following best practice recommendations for data screening (DeSimone et al., 2015), data were screened for quality prior to analysis. Of the 472 distributed surveys, 168 responses were removed based on the following criteria: (1) patterned answering (zero variance across more than 80% of items); (2) substantial missing data (>20% missing items); (3) completion time below 50% of the median; and (4) duplicate IP addresses with identical or near-identical response patterns (retaining only the first submission).

The exclusion rate was 35.6%. To assess whether this exclusion introduced bias, we compared the retained sample (*n* = 304) with the excluded cases (*n* = 168) for available demographic variables (gender, year, major program). We used chi-square tests to compare the excluded cases with the retained sample for critical observable factors (e.g., gender, year in school, program major). No significant differences were found (χ^2^ tests, *p* > 0.05), suggesting that the excluded cases were not systematically different from the retained sample with respect to observable characteristics.

#### 3.5.2 Quantitative analysis

The study’s analytical approach used Partial Least Squares Structural Equation Modeling (PLS-SEM) via SmartPLS 4 (Ringle et al., 2024). We selected PLS-SEM for three reasons. First, second-order constructs (need satisfaction and need frustration) are part of complex mediation pathways. Second, PLS-SEM is an appropriate method for exploratory theory testing in vocational education research (Hair et al., 2019, 2022). Third, it allowed us to account for indirect effects via bootstrapping procedures (Preacher and Hayes, 2008; Hayes and Little, 2022).

The model’s validity and reliability were assessed using the following criteria: (1) internal consistency reliability (Cronbach’s *α* and composite reliability ρc > 0.70); (2) convergent validity (average variance extracted [AVE] > 0.50); (3) discriminant validity (heterotrait-monotrait [HTMT] ratio <0.90; Henseler et al., 2015); and (4) multicollinearity (variance inflation factor [VIF] < 10).

Bootstrapping was conducted using 5,000 resamples, and a significance level of 0.05 was used to assess direct and indirect effects (Preacher and Hayes, 2008). Model fit was assessed using the Standardized Root Mean Square Residual (SRMR) with values below 0.08 indicating acceptable fit (Hair et al., 2022).

#### 3.5.3 Qualitative analysis

Qualitative data were analyzed using thematic analysis, a well-established method with a six-step procedure (Braun and Clarke, 2006). We performed multiple readings of the transcripts to identify trends in the data, followed by open coding to create initial labels for meaningful segments. We then grouped related codes into broader candidate themes, which we reviewed, combined, split, and refined until we had a clear, coherent thematic structure that captured the participants’ accounts. To perform a systematic management of the coding process, we used NVivo 14. In doing so, we maintained a dual orientation, which allowed us to identify trends as they emerged within the context of the SDT framework, thereby anchoring our findings in the participants’ voices without losing sight of the theoretical lens that informed the study (Fereday and Muir-Cochrane, 2006).

We established intercoder reliability following the approaches proposed by Campbell et al. (2013) to improve the reliability of our coding process. Two randomly selected researchers independently coded 6 of the 20 interviews (30% of the transcripts). Discrepancies in initial coding were resolved by consensus in meetings, and the coding strategy was iteratively modified during these meetings. The Cohen’s Kappa coefficient for the six transcripts was 0.84 (95% CI [0.78, 0.90]), showing strong intercoder agreement (Landis and Koch, 1977). Once the coding system was selected, the first investigator coded the remaining 14 transcripts.

We built codes iteratively by theme. First, initial codes were generated from line-by-line coding. Then, based on semantic similarity, the first codes were grouped into possible themes through axial coding. We then matched the emergent themes to the three essential psychological needs of SDT to ensure theoretical coherence. In this iterative manner, we arrived at three final themes, which we called Competence Seesaw, Constrained Autonomy, and Professional Relatedness. These themes aligned closely with the study’s theoretical framework (Braun and Clarke, 2006) and reflected a core experiential pattern in the data.

## 4 Results

### 4.1 Preliminary analyses

#### 4.1.1 Descriptive statistics

Table 3 exhibits the descriptive statistics for all constructs, including the means, standard deviations, and the Fornell–Larcker criterion for discriminant validity. All constructs demonstrated robust variability, with standard deviations ranging from 0.69 to 1.28. Need-supportive learning environment (NSLE) had the highest mean (M = 4.41, SD = 0.69), indicating that participants generally considered their English learning environment to be need-supportive. In contrast, the lowest mean scores were obtained for relatedness frustration (M = 1.76, SD = 1.04) and autonomy frustration (M = 2.38, SD = 1.28), indicating that cadets’ experiences of psychological need frustration were relatively infrequent.

**Table 3:** Descriptive statistics and Fornell-Larcker criterion for discriminant validity.

Variable	M	SD	1	2	3	4	5	6	7	8	9	10	11	12
1. NSLE	4.41	0.69	**(0.897)**											
2. Auto_S	4.30	0.76	0.821	**(0.951)**										
3. Auto_F	2.38	1.28	−0.155	−0.162	**(0.983)**									
4. Comp_S	4.27	0.77	0.735	0.693	−0.220	**(0.953)**								
5. Comp_F	2.34	1.25	−0.251	−0.163	0.549	−0.305	**(0.975)**							
6. Rel_S	4.26	0.77	0.637	0.619	−0.193	0.659	−0.222	**(0.974)**						
7. Rel_F	1.76	1.04	−0.342	−0.266	0.431	−0.333	0.501	−0.295	**(0.986)**					
8. ER	2.39	1.09	−0.261	−0.242	0.442	−0.271	0.493	−0.196	0.540	**(0.885)**				
9. INTR	2.62	1.08	−0.0131	−0.169	0.312	−0.175	0.397	−0.142	0.381	0.469	**(0.871)**			
10. IR	4.33	0.71	0.494	0.474	−0.258	0.558	−0.370	0.494	−0.335	−0.241	−0.136	**(0.907)**		
11. IM	3.84	0.95	0.443	0.447	−0.164	0.502	−0.371	0.481	−0.169	−0.144	−0.103	0.494	**(0.957)**	
12. ELO	4.17	0.73	0.553	0.528	−0.264	0.628	−0.457	0.594	−0.332	−0.256	−0.202	0.758	0.713	**(0.901)**

*N =* 304. Diagonal elements (in bold) are the square roots of AVE. All correlations with |r| > 0.113 are statistically significant at *p* < 0.05; those with |r| > 0.148 at *p* < 0.01.* Auto_F, Autonomy Frustration; Auto_S, Autonomy Satisfaction; Comp_F, Competence Frustration; Comp_S, Competence Satisfaction; ELO, English Learning Outcomes; ER, External Regulation; IM, Intrinsic Motivation; IR, Identified Regulation; NSLE, Need-Supportive Learning Environment; Rel_F, Relatedness Frustration; Rel_S, Relatedness Satisfaction.

#### 4.1.2 Measurement model assessment

##### 4.1.2.1 Discriminant validity

Discriminant validity was assessed using the Fornell–Larcker criterion and the HTMT ratio. As shown in Table 3, the square root of AVE for each construct (diagonal elements in bold) exceeded its correlation coefficients with other constructs, satisfying the Fornell–Larcker criterion (Fornell and Larcker, 1981). The HTMT values (Table 4) were all below the suggested threshold of 0.90, indicating robust discriminant validity (Henseler et al., 2015). The highest HTMT value (0.881 between NSLE and Autonomy Satisfaction) remained below the conservative threshold.

**Table 4:** HTMT values for discriminant validity assessment.

Construct	Auto_F	Auto_S	Comp_F	Comp_S	ELO	ER	IM	INTR	IR	NSLE	Rel_F	Rel_S
Auto_F												
Auto_S	0.174											
Comp_F	0.575	0.239										
Comp_S	0.176	0.773	0.331									
ELO	0.274	0.567	0.477	0.674								
ER	0.527	0.301	0.595	0.335	0.307							
IM	0.169	0.480	0.387	0.538	0.736	0.174						
INTR	0.336	0.178	0.429	0.184	0.202	0.592	0.112					
IR	0.270	0.519	0.393	0.611	0.797	0.292	0.519	0.148				
NSLE	0.160	0.881	0.262	0.786	0.570	0.311	0.456	0.130	0.520			
Rel_F	0.445	0.285	0.522	0.357	0.342	0.641	0.173	0.403	0.351	0.352		
Rel_S	0.202	0.673	0.235	0.714	0.621	0.237	0.503	0.146	0.526	0.664	0.308	

All HTMT values below 0.90 indicate satisfactory discriminant validity (Henseler et al., 2015).

##### 4.1.2.2 Reliability and convergent validity

All constructs demonstrated acceptable reliability and convergent validity. Table 2 shows that Cronbach’s *α* values ranged from 0.724 (External Regulation) to 0.970 (IM), all exceeding the 0.70 threshold. Composite reliability (ρc) values were all above the 0.70 threshold. All AVE values exceeded the 0.50 benchmark, supporting convergent validity (Fornell and Larcker, 1981).

##### 4.1.2.3 Model fit

The model’s overall fit was satisfactory, with an SRMR of 0.074. SRMR values below 0.08 are typically regarded as indicators of good model fit (Hair et al., 2022).

### 4.2 Hypothesis testing

#### 4.2.1 Direct effects

Our structural model revealed several significant direct effects (Table 5). The model explained 43.5% of the variance in English learning outcomes (ELO) directly from NSLE (*β* = 0.435, *p* < 0.001, 95% CI [0.331, 0.531]), with additional indirect effects bringing total R^2^ = 0.744 for learning outcomes.

**Table 5:** Direct effects.

Hypothesis	Effect	β	SE	t	*p*	Decision
H1a	NSLE → Autonomy Satisfaction	0.821	0.031	26.716	<0.001	Supported
H1b	NSLE → Competence Satisfaction	0.735	0.037	20.073	<0.001	Supported
H1c	NSLE → Relatedness Satisfaction	0.637	0.060	10.678	<0.001	Supported
H2a	Autonomy Satisfaction → Intrinsic Motivation	0.113	0.081	1.405	0.160	Not supported
H2b	Competence Satisfaction → Intrinsic Motivation	0.266	0.091	2.911	0.004	Supported
H2c	Relatedness Satisfaction → Intrinsic Motivation	0.231	0.103	2.242	0.025	Supported
H2a’	Autonomy Satisfaction →Identified Regulation	0.063	0.099	0.637	0.524	Not supported
H2b’	Competence Satisfaction →Identified Regulation	0.336	0.097	3.459	0.001	Supported
H2c’	Relatedness Satisfaction →Identified Regulation	0.182	0.117	1.560	0.119	Not supported
H4	Intrinsic Motivation → ELO	0.413	0.057	7.212	<0.001	Supported
H5	NSLE → Identified Regulation	0.079	0.110	0.722	0.470	Not Supported
H7a	NSLE → Autonomy Frustration	−0.155	0.057	2.716	0.007	Supported
H7b	NSLE → Competence Frustration	−0.251	0.051	4.880	<0.001	Supported
H7c	NSLE → Relatedness Frustration	−0.342	0.061	5.565	<0.001	Supported
H8a	Autonomy Frustration → External Regulation	0.168	0.076	2.209	0.027	Supported
H8b	Competence Frustration → External Regulation	0.216	0.077	2.793	0.005	Supported
H8c	Relatedness Frustration → External Regulation	0.337	0.067	5.044	<0.001	Supported
H8a’	Autonomy Frustration → Introjected Regulation	0.083	0.074	1.123	0.262	Not supported
H8b’	Competence Frustration → Introjected Regulation	0.241	0.079	3.042	0.002	Supported
H8c’	Relatedness Frustration → Introjected Regulation	0.232	0.069	3.356	0.001	Supported
H9a	External Regulation → ELO	−0.017	0.032	0.535	0.593	Supported
H9b	Introjected Regulation → ELO	−0.070	0.031	2.246	0.025	Supported

H2a’–H2c’ represent the sub-paths from need satisfaction to identified regulation; H8a’–H8c’ represent the sub-paths from need frustration to introjected regulation.

##### 4.2.1.1 Pathway 1: need satisfaction to autonomous motivation

NSLE was positively associated with autonomy satisfaction (*β* = 0.821, *p* < 0.001), competence satisfaction (*β* = 0.735, *p* < 0.001), and relatedness satisfaction (*β* = 0.637, *p* < 0.001). Thus, H1a, H1b, and H1c were supported.

For the need satisfaction into IM pathway, competence satisfaction (*β* = 0.266, *p* = 0.004) and relatedness satisfaction (*β* = 0.231, *p* = 0.025) were significant predictors, whereas autonomy satisfaction was not (*β* = 0.113, *p* = 0.160). Thus, H2b and H2c were supported, whereas H2a was rejected.

For the need satisfaction into IR pathway, competence satisfaction was a significant predictor (*β* = 0.336, *p* = 0.001), whereas autonomy satisfaction (*β* = 0.063, *p* = 0.524) and relatedness satisfaction (*β* = 0.182, *p* = 0.119) were not. Thus, H2b’ was supported, whereas H2a’ and H2c’ were rejected.

Intrinsic motivation positively predicted learning outcomes (*β* = 0.413, *p* < 0.001), supporting H4. However, NSLE did not significantly predict IR (*β* = 0.079, *p* = 0.470). Thus, H5 was rejected.

##### 4.2.1.2 Pathway 2: need frustration to controlled motivation

NSLE negatively predicted autonomy frustration (*β* = −0.155, *p* = 0.007), competence frustration (*β* = −0.251, *p* < 0.001), and relatedness frustration (*β* = −0.342, *p* < 0.001). Thus, H7a, H7b, and H7c were supported.

All three frustration dimensions significantly predicted external regulation, including autonomy frustration (*β* = 0.168, *p* = 0.027), competence frustration (*β* = 0.216, *p* = 0.005), and relatedness frustration (*β* = 0.337, *p* < 0.001). Thus, H8a, H8b, and H8c were supported.

For introjected regulation, competence frustration (*β* = 0.241, *p* = 0.002) and relatedness frustration (*β* = 0.232, *p* = 0.001) were significant predictors, though autonomy frustration was not (*β* = 0.083, *p* = 0.262). Thus, H8b’ and H8c’ were supported, whereas H8a’ was rejected.

External regulation did not significantly predict learning outcomes (*β* = −0.017, *p* = 0.593), supporting H9a. Introjected regulation had a small but statistically significant negative effect (*β* = −0.070, *p* = 0.025), supporting H9b.

#### 4.2.2 Indirect effects

Bootstrapping results with 5,000 resamples revealed significant indirect effects (Table 6). Competence satisfaction significantly mediated the NSLE-IM relationship (*β* = 0.195, *p* = 0.004, 95% CI [0.071, 0.336]), as did relatedness satisfaction (*β* = 0.147, *p* = 0.016, 95% CI [0.005, 0.241]). Thus, H3 was partially supported (H3a and H3b were supported; H3c was not supported).

**Table 6:** Indirect effects.

Hypothesis	Indirect path	β	SE	*p*	95% CI	Decision
H3a	NSLE → Comp_S → IM	0.195	0.068	0.004	[0.071, 0.336]	Supported
H3b	NSLE → Rel_S → IM	0.147	0.058	0.016	[0.005, 0.241]	Supported
H3c	NSLE → Auto_S → IM	0.093	0.071	0.162	[−0.043, 0.224]	Not supported
H6a	NSLE → Comp_S → IR → ELO	0.119	0.038	0.002	[0.052, 0.201]	Supported
H6b	NSLE → Rel_S → IR → ELO	0.056	0.036	0.115	[−0.022, 0.118]	Not supported
H6c	NSLE → Auto_S → IR → ELO	0.025	0.040	0.522	[−0.061, 0.094]	Not supported

The serial mediation path of NSLE → Competence Satisfaction → IR → ELO was significant (*β* = 0.119, *p* = 0.002, 95% CI [0.052, 0.201]), supporting H6a. However, the corresponding paths through relatedness satisfaction (*β* = 0.056, *p* = 0.115, 95% CI [−0.022, 0.118]) and autonomy satisfaction (*β* = 0.025, *p* = 0.522, 95% CI [−0.061, 0.094]) were not significant. Thus, H6b and H6c were rejected.

**Figure 2:**
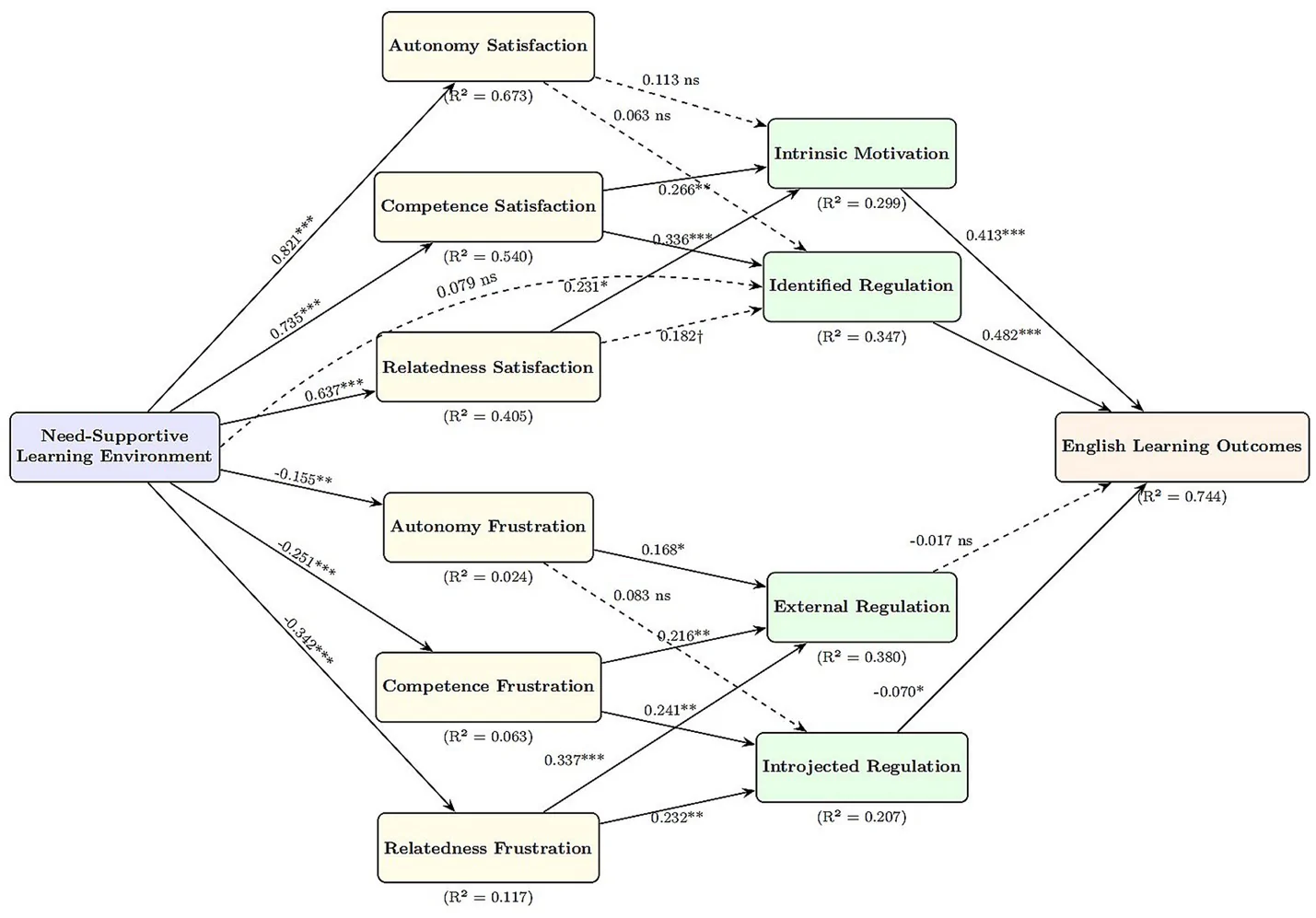
Structural model results with path coefficients (*β*) and *R*^2^ values.

The full structural model results, including path coefficients and R^2^ values, are presented in Figure 2.

### 4.3 Qualitative results

Three themes emerged from the thematic analysis of 20 in-depth interviews, each providing explanatory depth to the quantitative findings (Table 7). The analysis followed the six-phase procedure outlined by Braun and Clarke (2006), with intercoder reliability established at *κ* = 0.84 (see Section 3.5.3).

**Table 7:** Thematic coding structure with representative quotations.

Theme	Subthemes	Coding references (*n* = 20)	Representative quotation
Competence Seesaw	Academic confidence vs. operational anxiety	18	“I scored well on the listening test about engine room orders. But in the simulator, when the alarm sounds and the instructor role-plays a frantic chief engineer speaking quickly. my mind goes blank. I think, ‘Can I really do this on a real ship?’” (Second-year marine engineering cadet)
	Classroom mastery vs. real-world uncertainty		
Constrained Autonomy	Acceptance of mandatory certification	20	“We all know we must pass the test. It’s not a choice. But the way to prepare feels rigid. I’m aiming for cruise ships, so I want to practice more hospitality English, but our book only has dialogues about cargo ships. I wish we could choose some topics relevant to my own goals.” (Third-year navigation cadet)
	Desire for choice within fixed pathways		
	“Guided autonomy” over goal selection		
Professional Relatedness	Connection to maritime professional community	17	“When our teacher, a former captain, shares stories of using English during a storm or a port inspection, it does not feel like just a lesson. It feels like he’s preparing us for the brotherhood of seafarers.” (Second-year navigation cadet)

#### 4.3.1 Theme 1: the competence seesaw

This theme captures a common experiential pattern among cadets, a variable sense of competence that swung from the rigors of academic work to the intense operational contexts they encountered. This pattern illustrates the quantitative result that competence satisfaction was the strongest motivational predictor in our model. Cadets generally felt confident in the classroom, but this confidence often disappeared when they imagined—or faced—applying their knowledge to real-world context. Competence was the most salient psychological need amid the tension between academic performance and operational uncertainty in this safety-critical context. This dynamic was best exemplified by the story of one second-year marine engineering cadet:

“I passed the listening test on engine room orders well. But when the alarm goes off in the simulator, and the instructor plays the part of a frantic chief engineer speaking rapidly. My mind goes blank. And I think, Can I really do this on a real ship?”

This account highlights an important dissociation where success in formal assessments did not always correlate with perceived readiness for practice. The difference between “test-capable” and “operationally confident” helps to explain why competence satisfaction was so potent in its effects on cadets’ motivational outcomes.

#### 4.3.2 Theme 2: constrained autonomy

This theme provides insight into why IM was not significantly predicted by satisfaction of autonomy. Our qualitative data imply that cadets did not understand autonomy as absence of constraints, but as a matter of navigating constraints. All subjects agreed that the STCW Convention’s mandatory certification requirements were not open to discussion. However, within that fixed frame, cadets could distinguish between what they could not change (the pass requirement) and what they could influence (their preparation). Such “guided autonomy” involved selecting the method of learning, not the goals. A third-year navigation cadet summarized it as such:

“We all know we must pass the test. You cannot do it. But the way to get ready feels stiff. I am trying to practice more hospitality English. I am looking for a cruise ship, but our book only has dialogues about cargo ships. I’d like to be able to choose some topics that are relevant to my own goals.”

This suggests that autonomy support may be most appropriate in obligatory vocational contexts when it is embedded within fixed external requirements, allowing flexibility of means rather than ends.

#### 4.3.3 Theme 3: professional relatedness

The indirect path from relatedness satisfaction to learning outcomes via IR was weak and did not reach conventional statistical significance. We initially surmised that relatedness provided little impact; however, conversational data appeared less like typical peer bonding and more as a bridge to a future self. Cadets certainly valued their classmates, but the relatedness that had motivational import was forward-looking—toward the profession they hoped to enter and the community of seafarers they hoped to join. One second-year navigation cadet summarized it as such:

“It’s not like a lesson when our teacher, a former captain, tells stories about using English in a storm or during a port inspection. He seems to be training us for the brotherhood of the sea.”

That word “brotherhood” appears critical in implying that the participant shared a connection rooted in professional identity and a membership in a community that extends beyond the classroom, all of which take time to develop. Thus, a cross-sectional survey may capture specific timepoints but not the gradual process by which professional relatedness is internalized and transformed into motivation.

## 5 Discussion and implications

### 5.1 Interpretation of key findings

#### 5.1.1 Competence primacy in high-stakes vocational contexts

We found that competence satisfaction was the strongest driver of IM, whereas autonomy satisfaction had no marked effect. Though it was expected that standard SDT would result in the reverse outcome, or comparable effects across the two needs (Ryan and Deci, 2017; Vansteenkiste et al., 2020). This was not evident in our results.

The Competence Seesaw theme aided in deciphering this pattern. Cadets did not measure their capability against academic benchmarks, but rather against operational demands. For example, a cadet who aced their listening test might feel unprepared at the prospect of an engine room alarm. The ongoing tension between academic confidence and operational apprehension is the defining dynamic of vocational learning described by Billett et al. (2020)—students aren’t simply learning knowledge, they are transforming into a member who can be trusted with a ship and its crew.

In more conventional educational settings, competence and autonomy often correlate directly. However, maritime training is not considered a conventional setting, as cadets effectively evaluate themselves against two distinct standards simultaneously: the academic benchmark that measures what they know, and the operational benchmark that measures what they can do. Our data suggest that it is the latter that provides motivational weight. This primacy of competence is likely amplified by the safety-critical nature of maritime operations. Unlike many educational settings where skill deficits primarily affect grades, in maritime contexts competence gaps can have direct consequences for crew safety and vessel operations. The STCW certification framework explicitly codifies this reality by linking English proficiency to operational readiness, making competence not merely an aspirational goal but a professional necessity. This may explain why competence satisfaction carried more motivational weight than autonomy in this context—cadets are not simply learning for a grade, but for the ability to respond effectively under real-world operational pressures. Thus, cadets typically do not ask themselves “Did I get to choose?” but rather “If I were on a real ship right now, would I know what to do?”

Similar dissociations have been observed in vocational contexts, where cognitive load influences extrinsic motivation and self-efficacy influences IM (Zhang, 2024). Moreover, GenAI-IDLE activities were demonstrated to function through enhanced competence rather than increased choice (Cheng and Zhang, 2026), and AI chatbot-integrated writing tasks resulted in performance enhancements without corresponding improvements in self-efficacy (Gao et al., 2025). These results indirectly support the importance of competence satisfaction over skill signals and suggest that competence, not autonomy, motivates those who wish to work in skill-related fields.

#### 5.1.2 Constrained autonomy and the re-conceptualization of need support

Our results showed that autonomy satisfaction did not predict IM. This was expected according to SDT; however, these results do not suggest that autonomy is irrelevant in maritime VET. Rather, this indicates that the meaning of autonomy itself changes when certification is non-negotiable. The qualitative theme of Constrained Autonomy provides a direct explanation: cadets did not perceive autonomy as freedom from curricular requirements, but as meaningful choice within fixed structures. They accepted the STCW certification mandate as non-negotiable yet distinguished between what they could not change (the goal) and what they could influence (the pathway). This distinction—captured only through interviews—reveals that the null quantitative effect is not an anomaly but a reflection of how external mandates reshape the definition of autonomy in this context. Without the qualitative data, this finding might have been misinterpreted as a failure of SDT, when in fact it points to a contextual redefinition of the need itself. This integration thus illustrates how the mixed-methods design uncovers mechanisms that quantitative data alone cannot reveal.

*Guided Autonomy as a Contextual Redefinition*. Our results captured the contextual redefinition of guided autonomy among cadets. Cadets did not confuse autonomy with freedom from curricular requirements. Instead, they called it “guided autonomy,” wherein cadets had the ability to choose how and where to learn within a structure that could not change. This redefinition of autonomy explains why autonomy satisfaction showed no direct effect: once the goals are decided, autonomy is no longer about choosing the destination but about choosing the pathway.

This interpretation is supported by recent studies. Guo et al. (2026) found that effective maritime English teaching remains responsive to cadets’ communicative needs despite STCW-imposed syllabi, aligning with our notion of guided autonomy. Similarly, Zhang et al. (2026) showed that GenAI-mediated practice enhanced performance but not IM, suggesting that expanded learning pathways do not guarantee autonomy satisfaction when goals are externally imposed.

In theory, this finding builds on Vansteenkiste et al. (2020) discussion of contextual moderation in SDT, where it was theorized that independence is important within all contexts, but appears indifferent forms depending on the setting. Our data suggest a more specific proposition: in high-stakes vocational settings, autonomy support may be more productively operationalized, not as freedom from externally imposed requirements, but as meaningful choice within them. That distinction is important wherever external regulation is not a peripheral condition (e.g., STCW certification) but a defining feature of the professional landscape.

*Ceiling Effect on IR*. Although SDT generally predicts that a need-supportive environment facilitates internalization, this was not prominent in our data. This may be explained by a ceiling effect in which IR scores in our sample were remarkably high. Most cadets had already internalized the value of English for their professional futures, leaving little room for additional need support. In agreement, English proficiency is not optional for maritime cadets; it is a certification requirement built into the structure of their training. Zhang et al. (2025) observed a similar pragmatic orientation among vocational students more broadly, as they viewed learning as instrumental to professional goals rather than as an end unto itself.

If correct, the role of the need-supportive environment in our study is likely to be different from that which SDT would normally predict. Thus, rather than focus on internalizing new goals, it is more critical for cadets to maintain and deepen existing internalized goals, and stay motivated through the inevitable frustrations of training and the competing demands on their time and attention.

Together, the null autonomy–intrinsic link and the statistically non-significant NSLE–IR path points to a broader insight: when external certification is a provided, the motivational mechanisms SDT predicts in open educational settings operate differently. Autonomy support depends on means rather than ends, and need support functions less to drive initial internalization than to sustain already-internalized values.

#### 5.1.3 Professional relatedness as a distinct form of connection

Relatedness satisfaction predicted IM, but its indirect path through IR to learning outcomes was weak, just below the conventional threshold. However, data from interviews suggested a more complex scenario.

What cadets viewed as connection was not similar to the relatedness typically measured on SDT questionnaires (peer friendships, classroom camaraderie, being liked by classmates). Instead, they viewed it as such: “When he talks about stories of using English during a storm or a port inspection, it does not sound like just a lesson. It’s like he’s getting us ready for the brotherhood of seafarers.” Here, the word “brotherhood” suggests relatedness directed not to the present, but to the future and to a professional community that extended beyond peers. Thus, we coined the term “professional relatedness.”

The difference in relatedness is a distinction novel to SDT. The standard SDT formulation frames relatedness in terms of warmth and belonging in relationships, feeling connected to and cared for by others (Ryan and Deci, 2017). However, this definition is likely inadequate for describing vocational contexts. We found that relatedness also contains a professional dimension, which includes a sense of connection to the community cadets are preparing to enter and a sense of identification with that community as part of how they evolve. Guo et al. (2026) similarly found that professional identity mediates the relationship between social support and vocational students’ commitment to their craft. This suggests a broadening of relatedness that includes the people in the classroom and the external professional environment.

Note that professional identity develops relatively slowly and cannot be measured at distinct timepoints. This argument is supported by Billett et al. (2020), who showed that occupational identity is forged over months and years of training and workplace exposure. Therefore, a cross-sectional survey may not be able to characterize the gradual process by which professional relatedness feeds into IR and learning outcomes. Similar dynamics were found in work by Gao et al. (2025), where meaningful motivational change among vocational students required more time than a typical intervention window could capture. Our methodology allowed us to visualize this change despite the small result; it is therefore possible that, over a longer period and with a study design better adapted to capturing developmental processes, these changes would be markedly more evident.

#### 5.1.4 The frustration pathway and controlled motivation

All three forms of frustration predicted external regulation, and two of them—competence and relatedness frustration—also predicted introjected regulation. This pattern fits with the core logic of the dual-path framework, where satisfaction and frustration are not mirror images of one another but separate processes with distinct consequences (Howard et al., 2017).

The distinction between the two types of controlled motivation added another layer of nuance. External regulation (motivation driven by external contingencies such as exams or certification requirements) showed no detectable association with self-reported learning outcomes. Introjected regulation, by contrast, had a small but negative effect. This difference is noteworthy as introjected regulation is driven by internal pressures (guilt, shame, the need to maintain self-esteem) and appears to elicit a cost that external regulation does not. This aligns with core SDT principals, as not all forms of controlled motivation are equally problematic. External regulation can coexist with autonomous motivation when the external goals are themselves valued or internalized. Introjected regulation, however, introduces motivational friction that external regulation does not. The facilitative role of external feedback in promoting autonomous motivation has also been observed in other digital learning contexts. Hu (2025) found that interactive feedback on social media enhanced perceived competence, autonomy, and relatedness among adolescent English learners, which in turn fostered intrinsic motivation and improved learning outcomes. This suggests that when external inputs (such as feedback or certification requirements) are perceived as supportive rather than controlling, they can coexist with—and even reinforce—autonomous motivation.

Zhang et al. (2025) observed a similar pattern in their sample of vocational students. Here, controlled motivation predicted well-being, but the effect was weaker than that of autonomous motivation. Their findings, together with ours, suggest a more differentiated picture, one in which controlled motivation is not uniformly harmful, and specific forms are more corrosive than others. The practical implication is straightforward: if one is to understand how motivation works in vocational contexts, controlled motivation should not be treated as a single monolithic category. Instead, external and introjected regulation operate through distinct mechanisms and affect outcomes differently.

### 5.2 Theoretical contributions

This study extends the application of SDT to high-stakes vocational education by integrating need satisfaction and frustration, motivational regulation, and learning outcomes within a unified dual-path model, validated in the safety-critical, certification-bound context of maritime English training.

#### 5.2.1 Contextualized need hierarchy: competence as primacy

The first contribution extends SDT’s applicability by demonstrating a contextualized need hierarchy in regulated vocational settings. Contrary to SDT’s universalist assumption of equal contributions across domains (Vansteenkiste et al., 2020), our findings indicate that competence assumes primacy over autonomy when learners evaluate progress against occupational readiness rather than personal preference. This contextual weighting extends recent vocational L2 research reporting dissociations between performance gains and intrinsic motivation (Gao et al., 2025; Zhang et al., 2026) and likely applies to other safety-critical credential fields (e.g., aviation, medicine, military).

#### 5.2.2 Re-examining autonomy support: guided autonomy

While the underlying need for autonomy remains consistent with SDT’s predictions, the manifestation of autonomy support in this context takes a specific form—what we term guided autonomy. This does not represent a departure from SDT but rather a context-sensitive operationalization of autonomy support within externally constrained settings.

The second contribution we assessed concerns the theoretical tension in operationalizing autonomy support in fixed curricula. To bridge SDT the canonical definition of SDT (freedom to select goals and content; Reeve and Cheon, 2021) and the external certification mandates of high-stakes VET, we propose guided autonomy—agency over learning methods and pacing within prescribed boundaries—as an interpretive construct emerging from this specific maritime VET context, rather than a general theoretical extension. The null effect of autonomy-satisfaction, interpreted through the qualitative theme of Constrained Autonomy, suggests not irrelevance but contextual redefinition, where cadets wanted meaningful choice within requirements, not freedom from them. This reconceptualization extends the contextual moderation thesis of Vansteenkiste et al. (2020) and suggests that standard measures of autonomy (e.g., LCQ) may be insufficiently sensitive to agency in constrained professional contexts. It also represents methodological implication for future scale development.

#### 5.2.3 Expanding relatedness: introducing professional relatedness

The third contribution introduced professional relatedness as a contextualized extension of SDT’s interpersonal conceptualization of relatedness within maritime vocational training. We present this construct as an interpretation emerging from the present setting, while acknowledging that further research across diverse vocational contexts would be needed to assess its broader applicability. Unlike SDT’s emphasis on peer and instructor belonging (Ryan and Deci, 2017), professional relatedness operates through long-term occupational socialization and identity formation—a temporal dimension that accounts for the weak indirect effect of identified regulation in cross-sectional designs. This distinction supports a two-dimensional model of relatedness in VET (interpersonal vs. professional) that reconciles inconsistent mediation findings in prior vocational studies and aligns with identity-based perspectives of occupational commitment (Billett et al., 2020; Guo et al., 2026).

#### 5.2.4 Dual-path model validation and the differentiation of controlled motivation

The fourth contribution provides empirical validation of the dual-path SDT model in a safety-critical ESP context (maritime English training) and extends the theory’s treatment of controlled motivation. We argue that need satisfaction and need frustration operate through different pathways (Howard et al., 2017). To this end, we question the general assumption that controlled motivation is uniformly maladaptive. External regulation (associated with certification requirements) was not negatively related to self-reported outcomes, whereas introjected regulation (associated with internal shame) had a small but statistically significant negative effect. This subtype-specific distinction has been helpful in resolving conflicting findings in recent VET motivation research (Zhang et al., 2025) as the outcomes critically depend on which controlled-motivation subtype is involved, a nuance previously not considered.

Beyond these four substantive extensions, the sequential mixed-methods design itself offers a replicable template for investigating SDT’s boundary conditions in other externally regulated, non-WEIRD vocational contexts. The qualitative themes that emerged—competence seesaw, constrained autonomy, and professional relatedness—exemplify how this design can surface constructs that quantitative surveys alone cannot capture.

### 5.3 Practical implications

These findings have implications for practice at different tiers of the vocational education system. For students, the message is straightforward yet significant: test performance does not reflect work readiness, and academic success may misleadingly enhance self-confidence. Cadets who identify that gap and proactively pursue operational competency experiences are more inclined to remain engaged. These implications apply for professional relationships as well, where motivation derives from an increasing sense of affiliation with the profession for which one is preparing. For teachers, the implications manifest in three distinct directions. To allow cadets to authentically practice their skills and knowledge, teachers must develop more simulation-based mastery experiences, such as VR/AR drills that replicate bridge operations and emergency communications (Guo et al., 2026). Another approach is to provide substantial choices within the established curriculum: several courses through a unit, diverse case studies for distinct ship types, and alternative pace alternatives. This embodies our notion of “guided autonomy” (Reeve and Cheon, 2021). Another strategy is to engage alumni mariners as guest speakers or mentors to demonstrate to young cadets the importance of being part of the maritime community. At the institutional level, the implications are more structural. The curriculum must consider professional identity and linguistic proficiency. Student internships and industrial partnerships should transcend mere formalities into genuine professional experiences. Pedagogy must cultivate competence to meet demands and alleviate cognitive load to diminish dissatisfaction. For policymakers, communication pertains to facilitating situations. Teacher training should instruct educators on fostering autonomy within limitations. Assessment systems must reconcile certification with the advancement of competence, and simulation-based learning resources should be employed for bridging the academic-operational divide. These priorities are not novel, as they adhere to China’s Vocational Education Law and current quality initiatives (Zhang et al., 2025), as well as Han et al. (2023) call for digital transformation and professional development in vocational education. Our results offer empirical validation of these initiatives, and illustrate, from cadets’ viewpoints, the importance of these directives and the potential consequences of neglecting them.

### 5.4 Limitations and future directions

Our study had four distinct limitations.

First, we encountered causal inference. Our cross-sectional data cannot determine the direction of effects between need satisfaction, motivational regulation, and learning outcomes. Although SDT theory implies the hypothesized ordering, our results do not prove it. Longitudinal designs are needed, particularly to trace the gradual development of professional relatedness. Tracking cadets from pre-sea training through shipboard internships would allow researchers to model occupational identity internalization and test whether the competence-to-intrinsic pathway strengthens with authentic operational experience (Billett et al., 2020).

The second limitation concerns generalizability. Our sample was drawn from a single maritime college in Eastern China, leaving open the question of whether the observed need hierarchy holds across different settings. Comparative studies could examine between-country variation (e.g., cultural orientations, STCW implementation) and within-country variation across maritime specializations—navigation (bridge communication), marine engineering (technical manuals), and electro-technology (diagnostic protocols). Furthermore, the constructs of “guided autonomy” and “professional relatedness” introduced in this study should be viewed as interpretive frameworks emerging from this specific maritime VET context rather than as general theoretical extensions. Future research across diverse occupational training settings would be needed to assess their broader applicability and generalizability.

Third, our primary measures depended on self-reported data, increasing the likelihood of common-method bias. Although the qualitative component offers interpretive depth, future research may include objective indicators—such as proficiency test scores, behavioral logs, or instructor ratings—to ascertain if the identified motivational patterns forecast actual task persistence. Given that all principal variables were assessed through self-report measures, the high proportion of variance explained in learning outcomes should be interpreted with caution, as common method bias may have inflated the observed relationships. While the qualitative component provides some triangulation, future research incorporating objective indicators would help disentangle method variance from substantive effects. In addition to these enhancements, GenAI-mediated learning environments present a promising opportunity to expand the dual-path paradigm, since AI tools can concurrently undermine autonomy and bolster competence through adaptive feedback. Intervention studies may investigate if the contextual need weighting identified here applies to GenAI-supported learning and if guided autonomy serves as a design element for enhancing learner agency (Zhang et al., 2026).

Fourth, the data exclusion rate (35.6%) exceeded typical benchmarks. Bias checks revealed no systematic demographic differences between excluded and retained cases, though unmeasured traits (e.g., genuine motivation) may have differed. However, we acknowledge that the exclusion process may have disproportionately removed less engaged or less motivated respondents, as these unmeasured traits are often associated with inattentive responding. If so, the retained sample may be more homogeneous and potentially upward-biased in terms of motivation and engagement, which could affect the generalizability of the findings. This level of exclusion also raises the question of whether the final sample remains fully representative of the broader maritime cadet population, particularly regarding motivational dispositions. Future research could include attention checks and consider optimized survey designs to reduce inattentive responding while preserving adequate construct coverage.

Finally, the 12-item BPNSFS used in this study assigns only two items per subscale. While this version has shown adequate reliability (Chen et al., 2015), we acknowledge that using only two items to represent each psychological need substantially limits the breadth with which these constructs are captured. This is a notable limitation because autonomy, competence, and relatedness are central to the theoretical model. The reduced item pool may be particularly relevant for the marginal mediation observed through relatedness satisfaction, which could be partially attributable to measurement attenuation rather than a true null effect. This measurement limitation may be particularly relevant to the finding that competence emerged as the strongest predictor, as the narrower item coverage for each need could have attenuated the detection of more nuanced effects for autonomy and relatedness. Future research using the full 24-item version could clarify whether these weak indirect effects reflect genuine developmental dynamics or measurement attenuation.

Despite these limitations, our use of quantitative and qualitative data provides strong support for the contextual weighting of psychological needs in maritime VET. The pattern offers a useful point of departure for further research on high-stakes motivation in occupational English learning.

## 6 Conclusion

In this study, we aimed to characterize English-learning motivation among Chinese maritime cadets, using a sequential explanatory mixed-methods design that allowed us to perform a broad assessment via survey data in conjunction with qualitative data obtained through participant interviews. Three interrelated findings stand out.

First, the three basic psychological needs were not equally weighted across contexts. Competence was a much stronger motivator than autonomy, which had no direct effect on motivation. The Competence Seesaw theme explained why: cadets’ motivation was shaped not by choice but by the gap between academic success and operational readiness. Second, when goals are non-negotiable, autonomy support shifts from enabling goal choice to enabling choice of pathway and method—a principle we term “guided autonomy.” Third, relatedness in vocational settings extends beyond classroom peer relationships to include identification with the professional community cadets aspire to join, which we call “professional relatedness”—a connection that develops over time and resists capture in a single cross-sectional survey. The dual-path model further revealed that external regulation (driven by certification demands) did no detectable harm to learning outcomes, whereas introjected regulation (driven by internal shame) did small but measurable harm—a distinction that underscores the importance of treating controlled motivation subtypes separately.

The primary implication is not the quantity of student motivation but its nature. In certification-oriented settings, external mandates are non-negotiable; the challenge lies in facilitating students’ internalization of these requirements and helping them see them as essential milestones in their professional development. The suggested pathways—competence-building practice, guided autonomy within constraints, and connection to professional communities—provide concrete directions for educators, curriculum developers, and policymakers in vocational education.

## Data Availability

The raw data supporting the conclusions of this article will be made available by the authors, without undue reservation.
