# Supplementary material for: The psychological pathways to maritime English motivation: need satisfaction, frustration, and learning outcomes
Source: Front Psychol. 2026 Aug 24;17:1872269. doi: 10.3389/fpsyg.2026.1872269 (PMC13547243; doi:10.3389/fpsyg.2026.1872269)
Supplement: Supplementary file 1 [file Supplementary_file_1.docx]

**Appendix A: Questionnaire for Participants**

**Section 1: Demographic Information**

Gender: ( ) Male ( ) Female

Year of Study: ( ) Year 1 ( ) Year 2 ( ) Year 3

Major: ( ) Navigation ( ) Marine Engineering ( ) Marine Electro-Technology ( ) Other: __________

**Section 2: Survey Items**

**Instruction: Please indicate your level of agreement with the following statements regarding your English learning experience, using the scale:**

1 = Strongly Disagree, 2 = Disagree, 3 = Neutral, 4 = Agree, 5 = Strongly Agree

**A. Need-Supportive Learning Environment (NSLE)**

1.My English teacher listens to how I would like to do things.

2.My English teacher provides me with choices and options.

3.My English teacher encourages me to ask questions.

4.I feel understood by my English teacher.

5.My English teacher conveys confidence in my ability to do well in the course.

6.My English teacher tries to understand how I see things before suggesting a new way.

7.I am able to be open with my English teacher.

8.My English teacher handles students' emotions very well.

9.My English teacher clearly explains the meaning and purpose of learning tasks

**B. Basic Psychological Need Satisfaction and Frustration in English Learning (BPNSFS)**

**Autonomy Satisfaction**

10.I feel free to express my ideas in my English class.

11.I feel a sense of choice and freedom in the things I undertake in my English class.

**Autonomy Frustration**

12.Most of the things I do in English class feel like "I have to."

13.I feel forced to do many things in my English class that I wouldn't choose to do.

**Competence Satisfaction**

14.I feel confident that I can do well in my English class.

15.I feel capable at learning the maritime English required for my future job.

**Competence Frustration**

16.I have serious doubts about whether I can learn maritime English well.

17.I feel disappointed with many of my performances in English class.

**Relatedness Satisfaction**

18.I feel close and connected with other students in my English class.

19.I experience a warm feeling with the people in my English class.

**Relatedness Frustration**

20.I feel excluded from the group in my English class.

21.I feel that people in my English class are cold towards me.

**C. Motivation for Learning English**

**External Regulation**

22.I study English because I have to pass the mandatory maritime English exams.

23.I study English because it is a compulsory requirement of my maritime program.

24.I study English to avoid negative consequences (e.g., retaking courses) if my English is not up to standard.

25.I study English because my teachers and the school regulations expect me to do so.

**Introjected Regulation**

26.I study English because I would feel ashamed if I failed.

27.I study English because I would feel bad about myself if I didn't.

28.I study English because I want the teacher to think I'm a good student.

29.I study English to prove to myself that I am capable of handling the English challenges at sea.

**Identified Regulation**

30.Learning English is important to me for my future career as a seafarer.

31.It's important to me to learn English for my professional development.

32.I study English because it will help me get a good job on a ship.

33.I see learning English as relevant to my career goals.

**Intrinsic Motivation**

34.I study English because I enjoy it.

35.I study English because I think it's interesting.

36.I study English because I find it pleasurable to discover new things.

37.I study English for the enjoyment I experience when I grasp a difficult concept.

**D. English Learning Outcomes (ELO)**

38.My overall English proficiency has improved this semester.

39.My vocabulary related to maritime operations has increased.

40.My ability to understand English instructions (e.g., from a bridge simulator) has improved.

41.My confidence in speaking English in maritime scenarios has increased.

42.I actively participate and engage in my maritime English class.

43.I am satisfied with my progress in learning maritime English this semester.

44.I believe my current English level will help me pass future maritime certification exams.

45.I feel more prepared to use English in a real shipboard environment.

Section 3: Follow-up

46. Would you be willing to participate in a brief online interview (30-45 minutes) to further discuss your English learning experience? ( ) Yes ( ) No (If yes, please provide a contact email address): _________________________

**Appendix B: Sample Interview Questions**

1．Can you describe what a typical English class is like for you? What activities do you do?

2．Think about your motivation to learn English. What are the main reasons you put effort into it? Can you rank these reasons in order of importance?

3．(If career is mentioned): You mentioned your future career. Can you describe how learning English feels connected to becoming a seafarer? Does this connection make learning feel more meaningful or more like pressure?

4．Think about a time in your English class when you felt particularly capable and successful. What was happening? What did the teacher or your classmates do?

5．Conversely, can you describe a time you felt frustrated or ineffective in English learning? What led to that feeling?

6．How much influence do you feel you have over what you learn in English class (e.g., topics) and how you learn it? Can you give examples of choices you have or wish you had?

7．Do you feel a sense of connection or belonging with your classmates and teacher in the English class? How does this (or lack of it) affect your learning?

8．What kind of feedback do you receive on your English work? How does it make you feel? Does it help you understand your progress?

9．How do you perceive the relationship between passing the mandatory English tests/certifications and actually becoming a competent seafarer who can use English?

10．If you could change one thing about how maritime English is taught to make it more motivating for you, what would it be?
